# Platelet Autophagy as a Druggable Intracellular Pathway: Therapeutic Opportunities in Thromboinflammatory Diseases

**DOI:** 10.3390/pharmaceutics18030293

**Published:** 2026-02-27

**Authors:** Ting-Lin Yen, Jing-Shiun Jan, Ruei-Dun Teng, Pi-Chan Ko, Rajeev Taliyan, Chih-Hao Yang, Jui-Ming Sun, Joen-Rong Sheu

**Affiliations:** 1 Department of Pharmacology, School of Medicine, College of Medicine, Taipei Medical University, Taipei 110, Taiwan; d119096015@tmu.edu.tw (T.-L.Y.); d119101004@tmu.edu.tw (J.-S.J.); tang0803@tmu.edu.tw (R.-D.T.); chyang@tmu.edu.tw (C.-H.Y.); 2 Department of Medical Research, Cathay General Hospital, Taipei 106, Taiwan; 3 Graduate Institute of Medical Sciences, College of Medicine, Taipei Medical University, Taipei 110, Taiwan; 4 Department of Medical Research, Taipei Medical University Hospital, Taipei 110, Taiwan; 5 Section of Neurosurgery, Department of Surgery, Ditmanson Medical Foundation, Chia-Yi Christian Hospital, Chiayi City 600, Taiwan; 06494@cych.org.tw; 6 Neuropsychopharmacology Division, Department of Pharmacy, Birla Institute of Technology and Science-Pilani, Pilani Campus, Pilani 333031, Rajasthan, India; taliyanraja@gmail.com; 7 Research Center for Neuroscience, Taipei Medical University, Taipei 110, Taiwan; 8 Department of Nursing, School of Nursing, Fooyin University, Kaohsiung City 831, Taiwan

**Keywords:** platelet autophagy, drug targeting, intracellular therapeutics, thromboinflammation, pharmacological modulation, precision medicine, antiplatelet resistance

## Abstract

Platelet hyperreactivity is a central driver of thromboinflammatory diseases, including ischemic stroke, cardiovascular disorders, and autoimmune conditions. Current antiplatelet therapies primarily target surface receptors or coagulation pathways and are frequently limited by drug resistance, bleeding risk, and inadequate control of metabolically or inflammation-driven platelet dysfunction. Emerging evidence reveals that platelets possess a fully functional autophagic machinery that critically regulates mitochondrial quality, redox balance, granule secretion, cytoskeletal remodeling, and activation thresholds. This intracellular pathway represents a previously underrecognized but highly druggable regulatory axis in platelet biology. In this review, we examine the molecular framework governing autophagy in platelets, with emphasis on mTOR, AMPK, PI3K/AKT, and mitophagy signaling networks, and discuss how basal and activation-induced autophagy determine thrombotic behavior under physiological and pathological conditions. We then integrate clinical and preclinical evidence demonstrating how dysregulated platelet autophagy contributes to thrombotic risk in ischemic stroke, cardiovascular disease, metabolic disorders, and autoimmune diseases. Importantly, we highlight how pharmacological agents, including mTOR inhibitors, AMPK activators, natural autophagy enhancers, and lysosomal inhibitors, modulate platelet function through autophagy-dependent mechanisms. These findings position platelet autophagy as a promising intracellular therapeutic target that complements conventional antiplatelet strategies. We further discuss the translational challenges of autophagy-targeted therapy, including context dependency, lack of platelet-specific modulators, delivery strategies, and the need for reliable biomarkers to guide personalized intervention. By framing platelet autophagy as a druggable pathway rather than a biological curiosity, this review outlines a precision-targeted therapeutic framework for managing thromboinflammatory diseases through intracellular modulation of platelet behavior.

## 1. Introduction

Platelets, traditionally recognized as anucleate blood components responsible for hemostasis, have garnered increasing attention for their broader involvement in inflammatory responses [1], immunity [2], and thrombotic complications [3,4,5,6]. The finely tuned regulation of platelet activation is essential; its hyperactivation can lead to pathologic thrombus formation and vascular occlusion, whereas hypoactivation increases the risk of bleeding. Despite lacking nuclei, platelets retain a sophisticated set of intracellular signaling [7] and metabolic machinery [8,9,10] that enables them to maintain homeostasis and rapidly respond to vascular injury.

Clinically, platelet hyperreactivity is a central driver of thromboinflammatory diseases, including ischemic stroke, myocardial infarction, metabolic vascular complications, and autoimmune disorders. Current antiplatelet therapies, such as aspirin, P2Y12 receptor antagonists, and glycoprotein IIb/IIIa inhibitors, primarily target surface receptor signaling pathways [11]. While these strategies significantly reduce recurrent thrombotic events, important limitations remain. Inter-individual variability, high on-treatment platelet reactivity, pharmacogenomic influences, and drug resistance contribute to incomplete platelet inhibition in a substantial subset of patients. Moreover, intensified antiplatelet regimens are constrained by bleeding risk [12,13]. Notably, conventional agents largely suppress receptor-mediated activation but may not adequately address platelet dysfunction driven by metabolic stress, mitochondrial injury, oxidative imbalance, or inflammatory signaling [14,15], mechanisms increasingly recognized in chronic vascular and systemic inflammatory diseases.

Autophagy, a highly conserved intracellular process responsible for the degradation and recycling of cellular components, was once considered irrelevant in anucleate cells. However, a growing body of evidence has confirmed that platelets not only possess the autophagy machinery but also actively engage in autophagic processes [9,16,17]. These include the formation of autophagosomes [18], activation of mitophagy [16], and regulation of platelet granule content [19], all of which contribute to maintaining platelet viability, energy balance, and functional readiness.

In recent years, the modulation of autophagy has emerged as a critical determinant of platelet behavior, particularly in the context of thrombosis [20,21]. Studies using genetic [9,17,22] and pharmacological models [21,23] have demonstrated that both insufficient or excessive autophagy can alter platelet reactivity. These findings position autophagy as a novel intracellular regulatory axis that may complement conventional receptor-targeted strategies, particularly in diseases characterized by metabolic or inflammatory platelet dysregulation [24,25]. From a pharmacological perspective, targeting autophagy represents a mechanistically distinct strategy that complements traditional surface receptor antagonism by modulating intracellular stress response pathways governing platelet activation thresholds.

This review aims to provide a comprehensive overview of recent advances in understanding the role of autophagy in platelet biology, with particular emphasis on its contribution to thrombosis and related disease states. We examine the molecular architecture of autophagy in platelets, dissect its functional impact on activation and aggregation, and assess its relevance in thrombotic disorders such as ischemic stroke (IS), cardiovascular disease (CVD), and autoimmune conditions. Finally, we critically discuss the therapeutic potential and translational challenges of targeting autophagy as a precision strategy to modulate platelet-driven pathology.

### Literature Search Strategy

A comprehensive literature search was conducted using PubMed, Web of Science, and Scopus databases through December 2025. Search terms included “platelet autophagy,” “mitophagy in platelets,” “platelet thrombosis and autophagy,” “autophagy modulators,” “platelet storage lesion,” and “thromboinflammation.” Both preclinical and clinical studies were considered. Reference lists of selected articles were manually screened to identify additional relevant publications. Only English-language studies were included. Particular emphasis was placed on mechanistic investigations, genetic models, pharmacological interventions, and translational studies pertaining to thromboinflammatory diseases.

## 2. Molecular Machinery of Autophagy in Platelets

**Figure 1:**
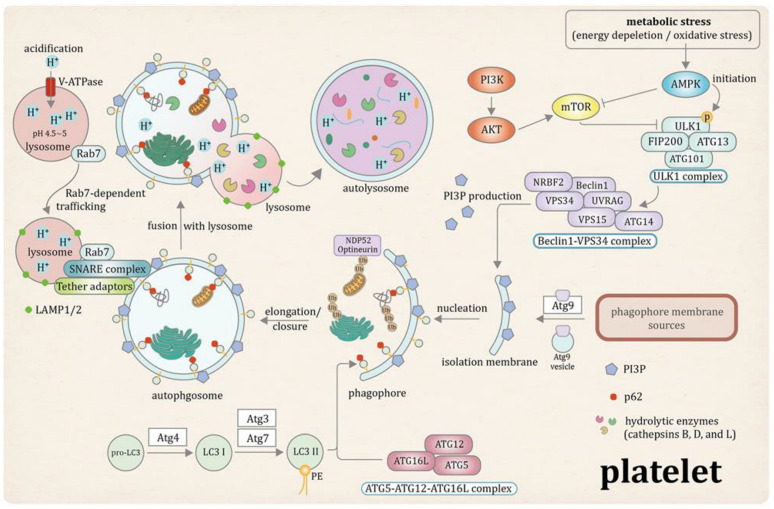
Molecular Machinery and Regulation of Autophagy in Platelets. This diagram outlines the core autophagy pathway in platelets, highlighting key molecular components involved in the initiation, nucleation, elongation, and degradation phases of autophagy. Upstream metabolic sensors, including AMPK and mTOR, regulate the ULK1 initiation complex (ULK1–ATG13–FIP200). Vesicle nucleation is mediated by the Beclin1–VPS34 complex, including VPS15 and ATG14L, leading to PI3P generation and phagophore formation. Autophagosome elongation requires the ATG5–ATG12–ATG16L1 conjugation system and LC3 processing (LC3-I to LC3-II) mediated by ATG4, ATG7, and ATG3. Selective cargo recognition involves adaptor proteins such as p62/SQSTM1. Fusion with lysosomes enables degradation of damaged organelles and oxidized substrates, thereby regulating platelet survival, activation, and functional responses under physiological and pathological conditions.

Despite their anucleate nature, platelets possess a remarkably intact and functional autophagy machinery. Far from being a passive or vestigial process, autophagy in platelets serves as a critical regulatory mechanism, supporting structural integrity [26], metabolic homeostasis [17], and activation potential [27]. Both human and animal studies have confirmed the presence of canonical autophagy pathways in platelets [28], including autophagosome formation [29], lysosomal degradation [23], and selective mitophagy [30]. These pathways actively modulate platelet function and contribute to their pro-thrombotic behavior under pathological conditions (Figure 1).

### 2.1. Core Autophagy Components Present in Platelets

Autophagosome biogenesis in platelets follows the conserved multi-step cascade described in nucleated cells, encompassing initiation, vesicle nucleation, membrane expansion, cargo recognition, and lysosomal degradation. However, in platelets, these molecular events operate under the specialized structural and metabolic constraints of short-lived circulating cells [31].

Autophagy initiation is governed by the ULK1 complex, composed of ULK1, ATG13, FIP200 (RB1CC1), and ATG101. In platelets, ULK1 functions as a metabolic sensor integrating upstream signals from mTOR and AMPK. Under nutrient-rich conditions, mTOR-mediated phosphorylation suppresses ULK1 activity, thereby inhibiting autophagy initiation [32]. Conversely, during energetic or oxidative stress, AMPK phosphorylates ULK1 at activating residues, promoting phagophore formation. Genetic disruption of ULK1 signaling in megakaryocyte-lineage cells impairs autophagic flux and compromises platelet aggregation and thrombus stability, confirming its functional relevance in platelet biology [17].

Following initiation, vesicle nucleation is mediated by the Beclin1–VPS34 class III phosphatidylinositol 3-kinase complex, which generates phosphatidylinositol-3-phosphate (PI3P) required for recruitment of downstream autophagy effectors to nascent phagophores [33,34]. This complex consists of the scaffold protein Beclin1, the catalytic lipid kinase VPS34, the regulatory adaptor VPS15, and accessory proteins including ATG14L, UVRAG, and NRBF2, which direct complex localization, maturation, and enzymatic activity. Deletion of *VPS34* in platelet-lineage cells markedly reduces PI3P production, impairs autophagosome formation, and disrupts platelet secretion and aggregation responses, underscoring the central importance of this complex in platelet autophagy.

Autophagosome elongation and closure require two ubiquitin-like conjugation systems. The ATG5–ATG12–ATG16L1 complex facilitates membrane expansion and curvature [35,36], while LC3 processing is mediated by sequential proteolytic and conjugation steps involving ATG4, ATG7, and ATG3. ATG4 cleaves pro-LC3 to generate cytosolic LC3-I, which is subsequently lipidated to LC3-II through ATG7- and ATG3-dependent reactions, enabling its integration into the autophagosomal membrane [37]. Efficient LC3 cycling is essential for dynamic autophagic flux during platelet activation. ATG9, the only multi-pass transmembrane ATG protein, contributes membrane delivery to the expanding phagophore; given the extensive membrane remodeling required during platelet activation and granule trafficking, ATG9-dependent membrane supply may be particularly relevant in these cells.

Importantly, platelet autophagy is not limited to bulk degradation but also includes selective cargo recognition. Adaptor proteins such as p62/SQSTM1 link ubiquitinated substrates to LC3 via LC3-interacting regions, enabling targeted sequestration of damaged proteins and organelles [22,38]. During mitophagy, additional receptors including NDP52 and optineurin facilitate recognition of ubiquitinated mitochondrial proteins. The degradation of p62 during platelet activation reflects active autophagic flux rather than passive protein turnover, supporting the presence of functional selective autophagy pathways in platelets.

Completion of autophagy requires fusion of autophagosomes with lysosomes to form autolysosomes. This process involves Rab7-dependent trafficking, SNARE-mediated membrane fusion, and lysosomal membrane proteins such as LAMP-1 and LAMP-2. Acidification of the autolysosomal compartment is mediated by vacuolar-type H^+^-ATPase (V-ATPase), enabling activation of lysosomal hydrolases including cathepsins B, D, and L, which execute cargo degradation [39,40,41,42]. Although platelet-specific mechanistic studies remain limited for this terminal phase, the established presence of lysosomal enzymes and functional degradative capacity supports the conclusion that platelet autophagy proceeds through a complete degradative cascade.

Collectively, these molecular components confirm that platelet autophagy adheres to the canonical autophagy architecture while functioning as a specialized intracellular quality-control system. Importantly, disruption of these core components in genetic models directly alters platelet activation, aggregation, and thrombotic potential, underscoring their pathological relevance (see Section 2.3).

### 2.2. Regulation of Platelet Autophagy by mTOR and AMPK Pathways

Similar to nucleated cells, autophagy in platelets is tightly regulated by nutrient- and energy-sensing pathways, notably the mechanistic target of rapamycin (mTOR) and AMP-activated protein kinase (AMPK), which modulate autophagic activity in response to metabolic cues.

**mTOR Pathway**: Serving as a central negative regulator of autophagy, mTOR activity under nutrient-rich conditions or chronic inflammation leads to the phosphorylation and inhibition of ULK1, thereby suppressing autophagy initiation [43]. In platelets, heightened mTOR signaling correlates with reduced autophagic activity and has been observed in patients with cardiovascular risk factors [44].

**AMPK Pathway**: Activated during energy depletion or oxidative stress, AMPK acts as a positive regulator of autophagy by phosphorylating ULK1 at distinct sites from those targeted by mTOR, thereby promoting autophagosome formation [45]. In platelets, AMPK activation is crucial for driving mitophagy and maintaining mitochondrial quality control, particularly under oxidative or metabolic stress conditions [46,47]. 

**PI3K/AKT Pathway**: Although primarily involved in platelet activation and survival, excessive activation of the PI3K/AKT pathway can suppress autophagy indirectly through mTOR signaling. Dysregulation of this pathway has been implicated in autoimmune platelet destruction and altered autophagic responses [48].

The dynamic interplay among these signaling pathways ensures that platelet autophagy is appropriately tuned to the cell’s energetic and metabolic demands, thereby preserving platelet function, and maintaining hemostatic balance.

### 2.3. Genetic Disruption of Autophagy and Its Impact on Platelet Function

Genetic models with megakaryocyte- or platelet-specific deletion of autophagy-related genes provide direct evidence that disruption of core autophagy machinery alters platelet function and thrombotic responses:

**Atg5 Deletion**: Mice with platelet-specific deletion of *Atg5* exhibit defective autophagosome formation, leading to impaired platelet aggregation, reduced granule secretion, and prolonged bleeding times. Notably, these mice demonstrate resistance to arterial thrombosis, underscoring the necessity of autophagy in thrombus formation [9].

**Atg7 Deletion**: Similarly, platelet-specific deletion of *Atg7* results in diminished LC3-II formation and a decreased LC3-II/LC3-I ratio, consistent with impaired autophagosome biogenesis. These mice display defects in platelet activation, further highlighting the importance of autophagy in platelet responsiveness [17].

**Beclin1 (Becn1) Heterozygosity**: Mice heterozygous for *Beclin1* (*Becn1*^+/−^) display impaired autophagic flux in platelets, accompanied by reduced responsiveness to agonists such as thrombin and collagen. These findings suggest that basal autophagy is critical for normal platelet reactivity and that its disruption leads to functional platelet abnormalities [22].

**VPS34 deletion**: Deletion of *VPS34* disrupts phosphatidylinositol 3-phosphate (PI3P) production, impairing basal autophagy and autophagosome formation. This defect leads to impaired platelet secretion and aggregation, and hampers the assembly of NADPH oxidase (NOX) complexes. These results underscore the critical role of VPS34-mediated autophagy in maintaining platelet functionality and regulating thrombotic responses [49].

Collectively, these genetic models offer compelling evidence that autophagy is essential for maintaining platelet functionality, governing their activation, granule secretion, aggregation, and contribution to thrombotic processes.

### 2.4. Mitophagy: Selective Mitochondrial Clearance in Platelets

Mitophagy, the selective autophagic removal of damaged or superfluous mitochondria, is a pivotal process of platelet autophagy and plays a critical role in maintaining cellular homeostasis [50]. Given that platelets rely heavily on mitochondrial metabolism to support its activation, calcium homeostasis, and Adenosine triphosphate (ATP) generation, maintaining mitochondrial integrity is essential. Under conditions of oxidative stress or hypoxia, mitophagy helps to preserve mitochondrial integrity and prevents excessive reactive oxygen species (ROS) production [51], thereby limiting platelet hyperactivation and sustaining hemostatic balance [9]. Several key regulators mediate mitophagy in platelets:

**PINK1–Parkin Pathway**: While extensively studied in neuronal contexts, components of the PINK1–Parkin pathway are also implicated in platelet mitophagy [47,52]. In healthy mitochondria, PINK1 is imported and rapidly degraded. However, upon mitochondrial depolarization or damage, PINK1 accumulates on the outer mitochondrial membrane, where it recruits the E3 ubiquitin ligase Parkin. Parkin ubiquitinates outer membrane proteins, marking the mitochondria for autophagic degradation.

**BNIP3 and NIX (BNIP3L)**: These mitochondrial outer membrane proteins act as mitophagy receptors, facilitating the removal of damaged mitochondria via interactions with LC3. In anucleate but active platelets, NIX is particularly important for mitochondrial clearance; its deletion leads to mitochondrial accumulation, heightened activation, and reduced platelet lifespan [53]. Although the role of BNIP3 in platelets is less well defined, its structural similarity to NIX suggests a compensatory or supportive role [54,55]. Together, NIX and BNIP3 ensure mitochondrial quality control and support both platelet production and hemostatic activity.

**FUNDC1**: FUNDC1 is a hypoxia-responsive mitophagy receptor that binds LC3 via its LC3-interacting region (LIR) to mediate mitochondrial degradation. In platelets, FUNDC1 is essential for mitochondrial quality maintenance, especially under stress conditions. Deficiency of *FUNDC1* impairs mitophagy, leading to mitochondrial accumulation, increased ROS generation, and heightened platelet reactivity, indicating its essential role in platelet homeostasis under stress conditions [56,57].

Importantly, these mechanistic pathways are not merely theoretical constructs but have been experimentally validated in disease-relevant contexts. For example, in diabetic models characterized by chronic oxidative stress, impaired mitophagy correlates with accumulation of dysfunctional mitochondria and enhanced platelet aggregation, linking defective autophagic quality control to prothrombotic phenotypes [47]. Similarly, in murine models of arterial thrombosis, genetic deletion of *VPS34* or *Atg7* disrupts autophagosome formation and results in attenuated thrombus stability under high-shear conditions [17,49]. These findings provide direct evidence that dysregulation of core autophagic machinery translates into altered thrombotic outcomes, thereby establishing pathological relevance beyond molecular signaling.

**Figure 2:**
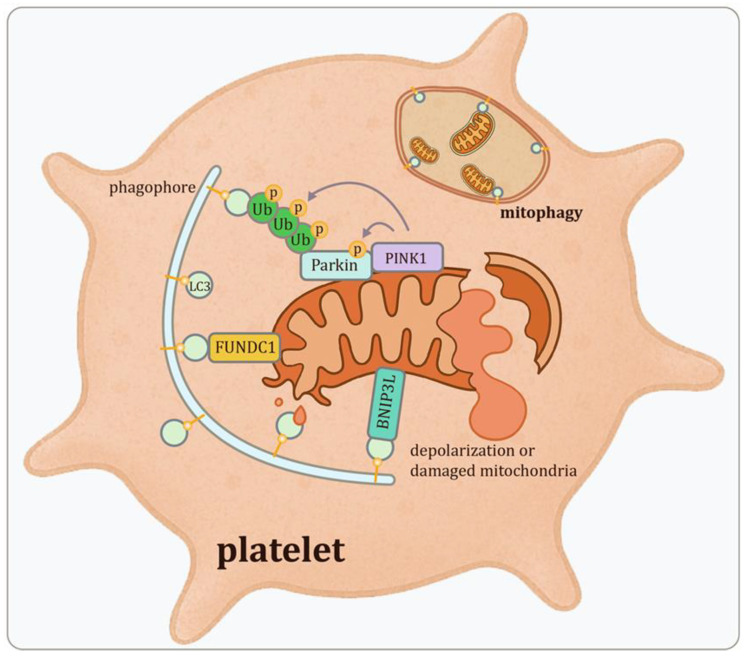
Mitophagy: Selective Mitochondrial Clearance in Platelets. This schematic illustrates the process of mitophagy in platelets, a selective autophagic pathway responsible for the removal of dysfunctional or damaged mitochondria. Upon mitochondrial depolarization or oxidative stress, PINK1 accumulates on the outer mitochondrial membrane and recruits the E3 ubiquitin ligase Parkin, which ubiquitinates various mitochondrial surface proteins. These ubiquitinated targets are recognized by autophagy receptors such as p62/SQSTM1, NDP52, and optineurin, which in turn interact with LC3 on the forming autophagosome membrane. The damaged mitochondrion is subsequently engulfed and delivered to the lysosome for degradation. This process ensures mitochondrial quality control, reduces ROS accumulation, and contributes to the regulation of platelet lifespan, reactivity, and thrombotic potential under stress conditions such as diabetes, aging, and ischemia.

Through these mechanisms (Figure 2), mitophagy serves a dual role in platelets: safeguarding mitochondrial integrity during cellular stress and preventing excessive platelet activation in pro-thrombotic environments.

### 2.5. Potential Contribution of Chaperone-Mediated Autophagy in Platelets

While macroautophagy and mitophagy represent the most extensively studied autophagic pathways in platelets, the potential involvement of chaperone-mediated autophagy (CMA) warrants consideration. CMA is a selective lysosomal degradation pathway in which cytosolic proteins containing KFERQ-like motifs are recognized by heat shock cognate protein 70 (HSC70) and translocated across the lysosomal membrane via LAMP-2A receptors [58]. Although direct experimental evidence for CMA activity in platelets remains limited, platelets possess functional lysosomes and protein quality-control machinery, suggesting that CMA-like mechanisms may contribute to the turnover of oxidatively modified or misfolded proteins under stress conditions [16,20]. Given the high metabolic turnover and oxidative burden experienced by circulating platelets, further investigation into CMA components such as LAMP-2A and HSC70 in platelet biology may reveal additional layers of intracellular regulation.

### 2.6. Assessment of Autophagy in Platelets

Assessing autophagy in platelets presents unique challenges due to their anucleate nature, small size, and limited organellar complexity. Nevertheless, several techniques have been successfully adapted to evaluate autophagic activity in these cells:

**Western Blotting**: Detection of autophagy-related proteins such as LC3, p62/SQSTM1, and Beclin1 offers molecular insights into autophagic flux [59]. Conversion of LC3-I (cytosolic form) to LC3-II (lipidated membrane-associated form) is commonly assessed, and the LC3-II/LC3-I ratio is frequently used as an indicator of autophagosome formation. However, accumulation of LC3-II may reflect either increased autophagosome biogenesis or impaired autophagosome degradation. Similarly, reduced p62 levels often suggest enhanced autophagic flux, but interpretation requires caution and ideally validation using lysosomal inhibitors to distinguish flux from static changes.

**Transmission Electron Microscopy (TEM)**: Regarded as the gold standard for autophagy assessment, TEM enables direct visualization of autophagosomes and autolysosomes within platelet cytoplasm [60]. It provides both qualitative and quantitative data on autophagic structures, though it is technically demanding and low-throughput.

**Flow Cytometry and Immunofluorescence**: These techniques enable the detection and quantification of autophagy-related proteins at the single-cell level. For instance, flow cytometry can be employed to measure LC3-II levels using specific antibodies [61], while immunofluorescence microscopy allows visualization of LC3 puncta formation, indicative of autophagosome accumulation and spatial distribution [62].

**Functional Assays with Pharmacological Modulators**: The use of autophagy inducers (e.g., rapamycin) or inhibitors (e.g., bafilomycin A1) can help elucidate the functional contribution of autophagy to platelet physiology [16,63]. Observing changes in platelet aggregation, granule release, ROS production, or lifespan in response to these agents provides indirect evidence of autophagic involvement.

Combining multiple methodologies enhances the reliability and interpretability of autophagy measurements in platelets, providing a more comprehensive understanding of how autophagy contributes to platelet function and disease-related phenotypes.

## 3. Autophagy as a Dual Regulator of Platelet Activation and Thrombosis

Autophagy, a fundamental process for degrading and recycling cytoplasmic components, plays a pivotal regulatory role in platelet biology [16]. In platelets, autophagy serves dual purposes: it supports activation under physiological conditions [17] and acts as a regulatory mechanism to prevent excessive reactivity under stress [27]. Unlike in most nucleated cells, where autophagy is primarily linked to stress responses and long-term adaptation, in platelets, this process dynamically regulates immediate activation processes, including cytoskeletal rearrangement [16,17,64], granule secretion [28], and mitochondrial homeostasis [53]. Recent evidence further suggests that basal autophagy maintains platelets in a metabolically balanced and activation-primed state [65], and that autophagic flux is rapidly modulated in response to external stimuli [66], indicating a more immediate role in platelet signaling dynamics than previously recognized.

### 3.1. Basal Autophagy Supports Platelet Activation Readiness

Resting platelets exhibit a constitutive level of autophagic activity that is believed to be essential for preserving their structural and metabolic integrity. This basal autophagy facilitates turnover of damaged proteins and organelles, compensating for the platelets’ limited protein synthesis capacity due to their anucleate nature. It supports organelle quality control [67,68], preserves granule contents [69], and maintains a balanced redox environment [70], all of which are essential for sustaining platelet viability and responsiveness. At the mitochondrial level, basal mitophagy prevents accumulation of depolarized mitochondria that would otherwise serve as persistent sources of superoxide and secondary ROS amplification. Excess mitochondrial ROS promotes calcium influx, phosphatidylserine exposure, and integrin αIIbβ3 activation, thereby lowering the threshold for procoagulant transformation [71,72]. By continuously clearing dysfunctional mitochondria, basal autophagy maintains platelets below this activation threshold and preserves redox-dependent signaling fidelity. Meanwhile, basal autophagy has been shown to regulate mitochondrial integrity, ATP production, and granule composition. These processes provide the metabolic support necessary for prompt platelet activation upon stimulation.

Genetic deletion of essential autophagy genes such as *Atg5* or *Atg7* results in reduced levels of LC3-II, accumulation of damaged mitochondria, and impaired clearance of cytoplasmic debris. Functionally, this leads to defective aggregation [16], diminished surface expression of activation markers including P-selectin and integrin αIIbβ3, and prolonged bleeding times in murine models [73].

These findings highlight the essential role of basal autophagy in maintaining the functional readiness of platelets and ensuring appropriate activation upon vascular injury.

### 3.2. Activation-Induced Autophagy: A Feedback Modulator

Beyond its basal housekeeping role, autophagy is dynamically modulated during platelet activation. Upon stimulation by physiological agonists such as thrombin, collagen, or ADP, platelets exhibit a rapid and transient increase in autophagic flux [9,27]. This is characterized by elevated conversion of LC3-I to LC3-II, reduced accumulation of the autophagy adaptor p62/SQSTM1, and increased formation of autophagosomes [74]. Although short-lived, this activation-induced autophagy is functionally significant and supports several key aspects of platelet activation.

**Facilitating degranulation and secretion**: Autophagy may support the trafficking and exocytosis of α- and dense granules by modulating membrane fusion events [75]. Autophagy-related proteins are thought to act as auxiliary machinery that supports the organization of membrane dynamics, enabling efficient granule mobilization [76].

**Regulating intracellular calcium and ROS levels**: Platelet activation is associated with a rapid rise in cytosolic calcium and ROS production. Autophagy, particularly via mitophagy, plays a protective role by removing dysfunctional mitochondria that are primary sources of excessive ROS and calcium dysregulation. Such process limits excessive ROS and calcium overload, thereby preventing mitochondrial-dependent platelet hyperactivation or damage [77].

**Recycling intracellular substrates for energy**: Platelet activation is a metabolically demanding process [78]. Autophagy aids in meeting this energy requirement by degrading and recycling cytoplasmic components into reusable substrates for ATP production [79]. This role is especially critical under nutrient deprivation or hypoxic conditions, where external energy sources are limited [80].

**Maintaining mitochondrial quality control**: Activation-induced autophagy preserves mitochondrial membrane potential, reduces oxidative damage, and prevents premature platelet exhaustion. Mechanistically, activation-induced autophagy appears to fine-tune the balance between energy production and procoagulant signaling. Platelet activation requires rapid ATP generation for actin polymerization, granule mobilization, and membrane remodeling [81,82]. However, excessive mitochondrial ROS during sustained activation can trigger apoptotic-like pathways and phosphatidylserine externalization. By dynamically adjusting mitophagic flux, autophagy prevents premature metabolic exhaustion while limiting ROS-driven hyperreactivity.

Taken together, activation-induced autophagy not only facilitates platelet responsiveness but also functions as a negative-feedback regulator. By supporting granule release, stabilizing intracellular signaling, and preserving bioenergetic reserves, autophagy ensures effective platelet activation while simultaneously preventing overactivation and premature exhaustion which ultimately safeguarding platelet function and lifespan.

### 3.3. Autophagy and Platelet Cytoskeletal Remodeling

Effective platelet activation requires dynamic cytoskeletal remodeling [83], including processes such as actin polymerization, shape change, pseudopodia formation, and membrane spreading. Emerging evidence suggests that autophagy directly contributes to these processes by coordinating intracellular trafficking and cytoskeletal organization through several key mechanisms:

**Interaction with the Actin Cytoskeleton**: Autophagy-related proteins such as LC3 and members of the ATG family have been shown to interact with actin-regulatory proteins [84,85,86]. In platelets isolated from *Atg5*- or *Atg7*-deficient mice, impaired autophagic flux is associated with defective actin reorganization and attenuated shape change upon thrombin stimulation. These defects correlate with reduced spreading capacity and diminished granule secretion, supporting a functional link between autophagic machinery and cytoskeletal rearrangement [18].

**Regulation of Integrin αIIbβ3 Function**: Autophagy also influences the surface expression and clustering of integrin αIIbβ3, a key receptor for fibrinogen binding during platelet aggregation. Genetic disruption of autophagy-related genes impairs integrin αIIbβ3 activation and membrane clustering [25], leading to reduced fibrinogen binding and compromised thrombus stability in ferric chloride–induced arterial injury models [60]. These in vivo findings demonstrate that autophagic dysfunction translates into impaired thrombus consolidation under shear stress conditions.

**Modulation of Platelet Spreading**: Autophagy appears to modulate platelet spreading on fibrinogen-coated surfaces, potentially by influencing actin polymerization and focal adhesion dynamics. Importantly, actin cytoskeletal remodeling is tightly coupled to intracellular ATP availability and localized calcium oscillations. Disruption of autophagy leads to mitochondrial dysfunction, reduced ATP production, and unstable calcium dynamics, impairing lamellipodia formation and spreading under shear stress. These defects translate into diminished thrombus consolidation in vivo, highlighting the structural-functional integration between autophagic flux and cytoskeletal mechanics [16].

These findings collectively demonstrate that autophagy is not merely a background metabolic process in platelets, but an active regulator of cytoskeletal and membrane dynamics. By influencing actin organization, integrin activation, and adhesion properties, autophagy fine-tunes platelet activation and aggregation during vascular injury. This mechanistic link underscores the multifaceted role of autophagy in modulating both the structural and functional aspects of platelet responses.

### 3.4. Pharmacologic and Genetic Evidence Supporting the Functional Role of Autophagy

Experimental studies employing genetic and pharmacological models have been instrumental in dissecting the multifaceted role of autophagy in platelet biology bridging its physiological functions with its implications in thrombotic pathology. These models have revealed how both the absence and dysregulation of autophagy can impact platelet behavior, bridging its physiological importance with its implications in pathological thrombosis.

**Pharmacological Modulation**: Chemical inhibition of autophagy using 3-methyladenine (3-MA) or bafilomycin A1 leads to impaired platelet aggregation, reduced P-selectin exposure, and diminished dense granule release [9]. These functional defects translate into attenuated thrombus formation in ex vivo and in vivo models. Conversely, pharmacological activation of autophagy with rapamycin enhances autophagic flux under basal conditions and sustains platelet responsiveness [64]. Notably, in models of oxidative stress or diabetes, rapamycin-mediated autophagy induction reduces platelet hyperreactivity and limits thrombus propagation [87], illustrating the context-dependent functional impact of autophagy modulation.

**Genetic Knockout Models**: Conditional deletion of autophagy-related genes in megakaryocyte-lineage cells provides definitive evidence of functional relevance. Platelets lacking *Atg7* [17,88] or *Beclin1* [89] exhibit defective aggregation, impaired spreading, and reduced thrombus stability in ferric chloride–induced arterial injury models. Similarly, *ULK1* deficiency results in compromised hemostatic responses and diminished thrombus consolidation under shear stress conditions [16,90]. These in vivo findings demonstrate that intact autophagic machinery is required not only for efficient platelet activation but also for stabilization of platelet-rich thrombi under pathological stress.

**Autophagic Flux Markers**: During platelet activation, increased LC3-II levels and p62 degradation confirm dynamic engagement of autophagic flux [91]. Importantly, modulation of these markers correlates with measurable changes in aggregation capacity and thrombus formation, supporting the concept that autophagy is functionally coupled to platelet reactivity rather than representing a passive cellular process.

Together, these findings provide direct mechanistic evidence that autophagy regulates platelet activity across both physiological and pathological contexts. By dissecting autophagy’s role using both genetic and chemical tools, these models illuminate how its modulation can either maintain hemostatic balance or contribute to thromboinflammatory disease, thereby setting the stage for understanding its behavior in systemic pathologies such as diabetes, atherosclerosis, and hypoxia-associated thrombosis.

### 3.5. Context-Dependent Modulation of Platelet Activation by Autophagy

Autophagy plays a dual and context-dependent regulatory role in platelet biology. While basal autophagy supports activation readiness under physiological conditions, it also functions as a critical safeguard that limits stress-induced platelet hyperactivation. Rather than operating as a unidirectional regulator, autophagy acts as a dynamic rheostat that adjusts platelet responsiveness according to metabolic and inflammatory cues. Consequently, its net effect on platelet-driven thrombosis depends on the nature, intensity, and duration of environmental stress.

Under conditions of oxidative stress, metabolic overload, or chronic inflammation, platelets experience mitochondrial dysfunction, excessive reactive oxygen species (ROS) production [92], calcium dysregulation [28], and bioenergetic instability [93], all of which promote hyperactivation and may trigger apoptotic-like pathways [94]. Through mitophagy-mediated clearance of damaged mitochondria, autophagy restores redox balance, stabilizes calcium signaling, and preserves mitochondrial function. In diabetic conditions, where platelets exhibit enhanced ROS production and a heightened prothrombotic phenotype, autophagy facilitates mitochondrial turnover and ROS detoxification, thereby reducing spontaneous aggregation and limiting exaggerated platelet reactivity [47]. These findings suggest a compensatory protective role of autophagy in chronic metabolic stress.

In contrast, during acute pathological stress such as hypoxia or inflammatory stimulation, platelet autophagy is markedly upregulated [56]. Genetic models provide strong evidence that autophagy is required for full platelet-driven thrombotic responses. Conditional deletion of autophagy-related genes such as *Atg5*, *Atg7*, or *Beclin1* in megakaryocyte-lineage cells impairs autophagosome formation and results in reduced platelet aggregation, secretion, and thrombus formation [73]. In murine arterial thrombosis models, including ferric chloride–induced vascular injury, these defects translate into delayed vessel occlusion, reduced thrombus stability, and prolonged occlusion times [18,49]. Importantly, these effects occur without significantly compromising baseline hemostasis, indicating that autophagy is particularly critical under high-shear or pathological stress conditions [95].

Pharmacological studies further support this context-dependent behavior. In acute hypoxic or ischemia–reperfusion settings, autophagy inhibition using agents such as chloroquine [96] or bafilomycin A1 [63] attenuates stress-induced platelet hyperactivity and reduces microthrombus formation [9,18]. Conversely, in chronic metabolic disease models such as diabetes and obesity, where sustained oxidative stress and mitochondrial dysfunction drive platelet hyperreactivity [97,98], pharmacological activation of autophagy using rapamycin [64] or spermidine [99] enhances mitochondrial quality control and reduces ROS accumulation [47], thereby dampening thrombotic propensity.

Collectively, these data demonstrate that autophagy does not function solely as a pro- or anti-thrombotic mechanism. Instead, its impact depends on the duration and intensity of stress, the underlying disease context, and the activation status of platelets. In chronic conditions characterized by persistent metabolic stress, such as diabetes, atherosclerosis [20], and systemic inflammation [60], enhancing autophagy may restore platelet homeostasis and reduce thromboinflammatory burden. In contrast, during acute thrombotic events or conditions of excessive autophagic activation (e.g., hypoxia-driven stress), transient inhibition may provide therapeutic benefit. Importantly, these mechanistic findings provide a biological explanation for the heightened thrombotic susceptibility observed in metabolic, inflammatory, and hypoxic disease states, suggesting that perturbations in platelet autophagic flux may represent a shared axis of thromboinflammatory risk.

**Figure 3:**
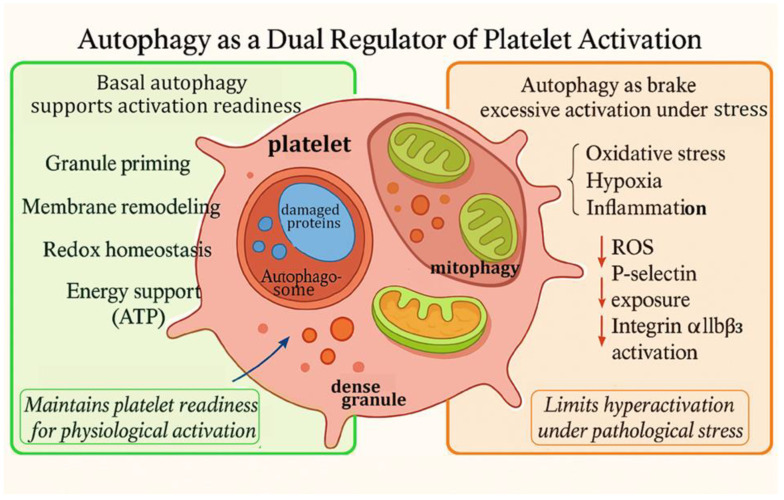
Context-dependent dual role of autophagy in platelet regulation. This schematic illustrates how autophagy dynamically modulates platelet activity across physiological and pathological contexts. Under basal conditions, autophagy preserves mitochondrial function, limits oxidative stress, and supports metabolic homeostasis, thereby maintaining platelet readiness for hemostatic responses. Under pathological stress, such as inflammation, hypoxia, or metabolic dysfunction, autophagy may either restrain hyperactivation through clearance of damaged organelles or, when excessively sustained, support platelet survival and thrombotic potential. The overall outcome depends on stress intensity, disease context, timing, and intrinsic autophagic capacity.

Therefore, precise understanding of disease environment, platelet stress status, and timing of intervention will be essential for safely translating autophagy-targeted strategies into thrombosis management. While preclinical evidence is compelling, further studies are required to define phase-specific therapeutic windows and to prevent unintended consequences such as bleeding or thrombotic rebound (Figure 3).

## 4. Disease Implications of Platelet Autophagy

**Figure 4:**
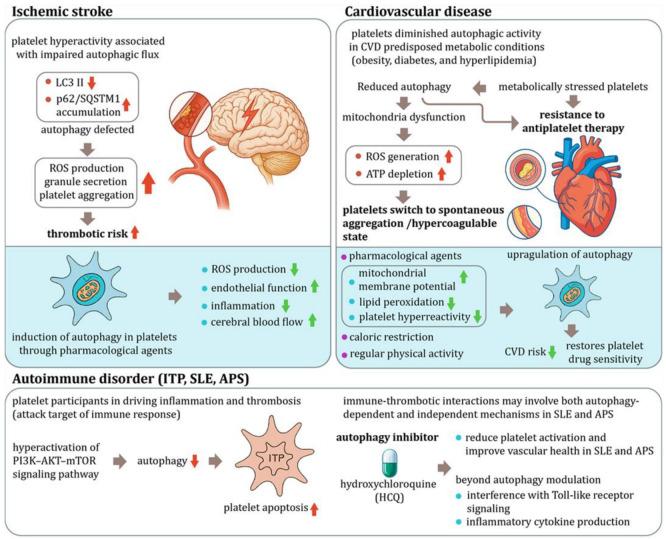
Disease-Specific Roles of Platelet Autophagy: Mechanisms and Pathological Impact. Platelet autophagy exhibits distinct functional roles across major disease categories. In metabolic and thrombotic disorders, impaired autophagy leads to increased oxidative stress and platelet hyperactivity. In autoimmune conditions like ITP, autophagy supports platelet survival, whereas in SLE and APS, its dysregulation may amplify immune-mediated platelet activation. During acute ischemic stroke and hypoxia, autophagy shows a temporal dual role, contributing to early thromboinflammation while supporting vascular recovery in later phases.

The regulation of platelet function by autophagy is not merely a cellular curiosity but a biologically significant mechanism with far-reaching implications in human diseases. Emerging evidence has linked dysregulated platelet autophagy to the pathogenesis of several major conditions, including ischemic stroke, cardiovascular disease, and autoimmune disorders (Figure 4). Alterations in autophagic activity affect key aspects of platelet physiology, such as mitochondrial quality control, granule secretion, and activation thresholds, which in turn influence thrombotic and inflammatory outcomes.

This section explores the role of altered platelet autophagy in the progression of these diseases. We highlight how impaired or excessive autophagic activity contributes to disease-specific platelet dysfunction and examine the potential of targeting autophagy pharmacologically as a therapeutic strategy in each context.

### 4.1. Ischemic Stroke

Ischemic stroke, characterized by the occlusion of cerebral arteries and subsequent oxygen deprivation in brain tissue, is a thrombotic and inflammatory event in which platelet hyperactivity plays a pivotal role. In individuals with underlying risk factors such as metabolic syndrome [100], atrial fibrillation [101], or chronic inflammation [102,103], platelets often exhibit hyperactivity associated with impaired autophagic flux.

Clinical studies have shown that platelets from stroke-prone patients exhibit reduced levels of LC3-II [104], a marker of autophagosome formation and increased accumulation of p62/SQSTM1, indicating defective autophagy [18,105]. This impairment correlates with elevated ROS production, increased granule secretion, and enhanced platelet aggregation, all of which contribute to heightened thrombotic risk.

Pharmacological strategies aimed at restoring autophagic activity have demonstrated therapeutic potential in mitigating these prothrombotic platelet responses: Trehalose, a natural disaccharide, has been shown to reduce oxidative stress and suppress platelet activation [21]. Spermidine, a polyamine that enhances autophagy, spermidine has been reported to decrease platelet aggregation and ROS production [8]. Nicotinamide, a form of vitamin B3, enhances mitochondrial quality control by restoring autophagic flux, thereby reducing platelet oxidative burden [106,107].

In vitro studies using these agents have confirmed their ability to restore platelet autophagy and reverse hyperreactivity. Supporting evidence from preclinical stroke models further strengthens the rationale for autophagy-targeted therapies. By using the spontaneously hypertensive stroke-prone rats (SHRSP), administration of trehalose in these rats reduced stroke incidence and renal injury [108], suggesting a protective role of autophagy activation. Similarly, studies utilizing the rodent stroke model of middle cerebral artery occlusion (MCAO), treatment with spermidine [109] or nicotinamide [110] led to decreased infarct volumes and improved neurological outcomes.

The beneficial effects of autophagy activation in ischemic stroke are attributed to multiple mechanisms:

**Reduction in Oxidative Stress**: Autophagy facilitates the removal of damaged mitochondria, thereby decreasing ROS production and preventing oxidative damage to platelets and vascular endothelial cells [111].

**Support of Endothelial Function**: By removing damaged cellular components, autophagy enhances endothelial cell health and vascular reactivity, promoting better cerebral perfusion [112].

**Suppression of inflammation**: Autophagy attenuates inflammatory signaling by reducing pro-inflammatory cytokine production and limiting the expression of adhesion molecules that facilitate leukocyte–endothelium interactions [113].

**Improvement in cerebral blood flow**: By controlling platelet activation during the vulnerable post-ischemic period, autophagy helps prevent microvascular obstruction and supports reperfusion [114].

These findings support the concept that enhancing platelet autophagy may represent a viable strategy for stroke prevention in high-risk individuals. However, in the acute stroke setting, where rapid clot dissolution is critical, the timing and safety of autophagy-targeting interventions remain to be fully defined and warrant further investigation.

### 4.2. Cardiovascular Diseases

Cardiovascular diseases (CVDs), including coronary artery disease, myocardial infarction, and peripheral artery disease, are closely associated with platelet hyperreactivity, oxidative stress, and endothelial dysfunction. Accumulating evidence suggests that autophagy is a key regulatory mechanism modulating platelet behavior in the pathological setting of these disorders [25].

In metabolic conditions that predispose to CVD, such as obesity [115], diabetes, and hyperlipidemia [116], platelets frequently exhibit diminished autophagic activity, contributing to a range of dysfunctions:

**Mitochondrial Dysfunction**: Reduced autophagy compromises mitochondrial turnover, leading to the accumulation of dysfunctional mitochondria, increased ROS generation, and ATP depletion [117]. This metabolic instability predisposes platelets to spontaneous aggregation and contributes to a hypercoagulable state [118]. 

**Resistance to Antiplatelet Therapy**: Suppressed autophagy in metabolically stressed platelets may underlie reduced responsiveness to antiplatelet agents such as aspirin and clopidogrel, complicating the management of thrombotic risks in CVD patients [119,120]. While the exact mechanisms remain to be fully defined, impaired intracellular stress adaptation and mitochondrial quality control may alter platelet drug sensitivity and survival, thereby affecting the therapeutic efficacy of antiplatelet drugs.

Experimental studies in diabetic mouse models have demonstrated that enhancing autophagic flux, particularly mitophagy, restores mitochondrial membrane potential, reduces ROS production, and limits lipid peroxidation [121]. These improvements lead to better platelet responsiveness to physiological stimuli without significantly increasing bleeding risk [27,47]. Additionally, pharmacological agents that promote autophagy have demonstrated benefit in reducing thrombotic risk in CVD models: AMPK Activators (e.g., Metformin): By activating AMP-activated protein kinase (AMPK), metformin enhances autophagy, improving mitochondrial function and reducing oxidative stress, not only in platelets but also in vascular endothelial cells [122]. mTOR Inhibitors (e.g., Rapamycin): Inhibition of the mechanistic target of rapamycin (mTOR) pathway by rapamycin has been demonstrated to induce autophagy, leading to improved metabolic profiles and reduced platelet hyperreactivity in diabetic models [123].

Given the chronic nature of cardiovascular diseases, long-term interventions that upregulate autophagy, such as caloric restriction [124] and regular physical activity [125] have been shown to upregulate autophagy, potentially contributing to improved cardiovascular health and reduced thrombotic risk.

In summary, impaired autophagy contributes to platelet dysfunction and thrombotic risk in cardiovascular diseases, especially in the context of metabolic stress. Pharmacological and lifestyle-based modulation of autophagy offers a promising therapeutic avenue, but further clinical validation is needed to evaluate the safety, efficacy, and timing of such interventions in CVD patients.

### 4.3. Autoimmune Disorders

In autoimmune conditions such as immune thrombocytopenia (ITP) [126], systemic lupus erythematosus (SLE) [127], and antiphospholipid syndrome (APS) [128], platelets act as both platelets are not only victims of immune attack but also active participants in driving inflammation and thrombosis. Autophagy, as a regulator of cell survival, immune signaling, and metabolic homeostasis, plays a crucial and context-dependent role in these diseases, offering potential therapeutic avenues for immune modulation and thromboinflammatory control.

**Immune Thrombocytopenia (ITP)**: ITP is characterized by autoantibody-mediated destruction of platelets and impaired platelet production, resulting in thrombocytopenia and increased bleeding risk. Emerging evidence indicated that platelets from ITP patients exhibit reduced autophagic activity, which has been linked to hyperactivation of the PI3K–AKT–mTOR signaling pathway [64]. This autophagy deficiency contributes to accelerated platelet apoptosis, as evidenced by increased Annexin V binding and mitochondrial depolarization.

In vitro studies have demonstrated that pharmacological activation of autophagy using rapamycin can restore autophagic flux, decrease platelet apoptosis, and enhance platelet survival. Clinically, low-dose rapamycin has shown therapeutic benefit in refractory ITP, with response rates of 70% at 6 months and 65% at 12 months [129], supporting the idea that autophagy enhancement may improve platelet lifespan and count in this disorder.

**Antiphospholipid Syndrome (APS) and Systemic Lupus Erythematosus (SLE)**: In APS, autoantibodies, especially antiphospholipid antibodies (aPL) promote platelet activation, endothelial dysfunction, and thrombosis [130]. The antimalarial agent hydroxychloroquine (HCQ), a common treatment for SLE and APS, has been shown to reduce platelet activation and improve vascular health [131]. Studies have demonstrated that HCQ use in SLE patients is associated with decreased platelet aggregation and downregulation of platelet activation-related transcripts. Furthermore, HCQ has been linked to improved endothelial function, suggesting its role in mitigating thrombosis risk in APS and SLE patients.

Interestingly, while HCQ is a well-known autophagy inhibitor, its antithrombotic effects in APS and SLE may involve mechanisms beyond autophagy modulation, including interference with Toll-like receptor signaling and reduction in inflammatory cytokine production [132]. These findings highlight the complexity of autophagy’s role in autoimmune disorders and underscore the need for context-specific therapeutic approaches. 

In summary, autophagy plays context-dependent and divergent roles in autoimmune disorders. In ITP, enhancing autophagy supports platelet survival and reduces apoptosis, whereas in APS and SLE, modulation of immune-thrombotic interactions may involve both autophagy-dependent and independent mechanisms. These findings underscore the importance of disease-specific and pathway-targeted strategies when considering autophagy as a therapeutic target. Future studies are needed to clarify the precise molecular pathways by which autophagy intersects with immune dysregulation, platelet activation, and thrombotic risk in autoimmune conditions.

## 5. Autophagy-Based Therapeutic Strategies for Platelet Dysfunction

Given its central role in regulating platelet activation, survival, and thromboinflammatory behavior, autophagy has emerged as a promising therapeutic target across a range of disease contexts. Unlike conventional antiplatelet therapies that primarily act on surface receptors or coagulation pathways, autophagy-based strategies offer a novel intracellular approach in modulating platelet bioenergetics, redox balance, and activation thresholds at the metabolic and organelle levels.

This intracellular mode of action makes autophagy modulation particularly appealing in conditions where platelet dysfunction stems from metabolic stress, oxidative injury, or immune dysregulation. However, due to the context-dependent nature of autophagy, therapeutic modulation requires careful calibration to avoid exacerbating platelet dysfunction or increasing bleeding risk. The following sections explore current pharmacological modulators of platelet autophagy, highlight candidate interventions, and discuss future directions for precision-targeted autophagy-based therapies in thrombotic and inflammatory diseases.

### 5.1. Pharmacological Modulators of Platelet Autophagy

A number of clinically approved or experimental agents with autophagy-modulating properties have been evaluated for their effects on platelet function and thrombotic behavior (summarized in Table 1). These compounds broadly fall into two categories: autophagy activators and autophagy inhibitors, each exhibiting context-dependent therapeutic potential.

Autophagy activators such as rapamycin, metformin, spermidine, and resveratrol enhance autophagic flux through distinct upstream mechanisms, including mTOR inhibition or AMPK activation. In preclinical models, these agents improve mitochondrial quality control, reduce oxidative stress, and attenuate platelet hyperreactivity, particularly in metabolic and inflammatory disease settings [27,129]. Their beneficial effects appear most consistent in chronic conditions characterized by sustained oxidative stress and mitochondrial dysfunction, such as diabetes or immune-mediated disorders [133,134,135].

Conversely, autophagy inhibitors including chloroquine, hydroxychloroquine, and bafilomycin A1 suppress lysosomal acidification [96] or autophagosome–lysosome fusion [136]. In acute stress models, particularly hypoxia or inflammatory stimulation, autophagy inhibition reduces platelet aggregation and thrombus formation [18]. However, most evidence derives from experimental systems, and platelet-specific effects remain difficult to disentangle from systemic immunomodulatory actions.

Importantly, the therapeutic efficacy of autophagy modulation appears highly dependent on disease context, timing, and magnitude of intervention. Chronic enhancement of autophagy may restore platelet homeostasis in metabolic disorders, whereas transient inhibition may be beneficial in acute thrombotic states. Nevertheless, systemic autophagy modulation carries risks, including impaired immune responses, platelet exhaustion, thrombotic rebound, or bleeding complications. These limitations highlight the need for platelet-targeted delivery strategies, phase-specific intervention, and reliable biomarkers to guide precision therapy.

### 5.2. Disease-Specific Therapeutic Strategies Targeting Platelet Autophagy

**Figure 5:**
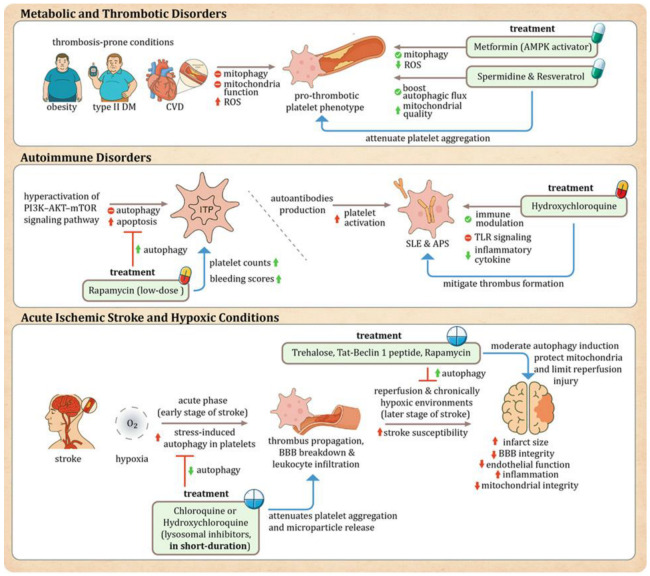
Disease-Specific Therapeutic Approaches Targeting Platelet Autophagy. Autophagy-targeting strategies are tailored to distinct disease settings. In metabolic and thrombotic disorders, autophagy inducers (e.g., metformin, spermidine) restore mitophagy and reduce oxidative stress, mitigating platelet hyperactivity. In ITP, agents like rapamycin enhance autophagy to preserve platelet survival. Conversely, in SLE and APS, autophagy inhibitors such as hydroxychloroquine reduce platelet-driven inflammation. In ischemic stroke and hypoxia, timing is critical, autophagy inhibition during the acute phases reduces thromboinflammation, while activation during recovery promotes mitochondrial integrity and vascular repair.

Given the context-dependent role of autophagy in platelet biology, therapeutic strategies must be tailored to the disease setting, stage, and duration of stress. Unlike conventional antiplatelet agents that broadly suppress platelet activation, autophagy modulators offer a potentially more nuanced intracellular approach to restoring platelet homeostasis when applied with temporal and pathological precision (Figure 5).

#### 5.2.1. Metabolic and Thrombotic Disorders

In metabolic syndrome, type 2 diabetes, and coronary artery disease, platelets are chronically exposed to low-grade inflammation and oxidative stress [137], leading to mitochondrial dysfunction and a sustained prothrombotic phenotype [138,139,140]. In these conditions, impaired mitophagy contributes to excessive ROS accumulation and platelet hyperreactivity.

Pharmacological activation of autophagy, using agents such as metformin, spermidine, or resveratrol, has demonstrated beneficial effects in preclinical models by restoring mitochondrial quality control and reducing oxidative stress [21]. Metformin improves platelet mitochondrial membrane potential and normalizes reactivity in diabetic models, while spermidine [141] and resveratrol enhance autophagic flux and attenuate oxidative injury [142]. These findings suggest that autophagy enhancement may reduce thrombotic risk in chronic metabolic disorders, particularly in patients exhibiting resistance to conventional antiplatelet therapies.

Notably, although mechanistic data in diabetic and metabolic models are compelling, clinical studies directly measuring platelet autophagic flux in large patient cohorts remain scarce. Thus, whether autophagy restoration translates into durable antithrombotic benefit in humans requires further investigation.

#### 5.2.2. Autoimmune Disorders

In immune thrombocytopenia (ITP), autophagy serves a cytoprotective role by preventing platelet apoptosis. Suppressed autophagic activity in ITP platelets [16] contributes to mitochondrial instability and reduced survival. Pharmacological activation of autophagy using rapamycin or synthetic compounds such as ABO restores LC3-II levels, reduces p62 accumulation, and improves platelet viability in vitro [64]. Clinical observations indicate that low-dose rapamycin may increase platelet counts in refractory ITP patients [129], supporting the translational relevance of autophagy induction in this setting. However, available clinical evidence is based on relatively small cohorts and has not been specifically designed to dissect platelet-autophagy–dependent mechanisms, leaving uncertainty regarding the extent to which therapeutic benefit derives from direct platelet effects versus broader immunomodulatory activity.

In systemic lupus erythematosus (SLE) and antiphospholipid syndrome (APS), the therapeutic landscape differs. Hydroxychloroquine (HCQ), a lysosomal inhibitor, reduces thrombotic risk and platelet activation signatures in SLE patients [131]. Although HCQ impairs autophagic flux, its antithrombotic effects likely arise from broader immunomodulatory mechanisms, including Toll-like receptor inhibition and cytokine suppression. These contrasting strategies underscore the necessity of disease-specific modulation: autophagy activation to preserve platelet survival in ITP, versus selective inhibition or immune-targeted modulation in autoimmune thromboinflammatory states.

#### 5.2.3. Acute Ischemic Stroke and Hypoxic Conditions

Acute ischemic stroke and hypoxia present a temporally dynamic environment in which autophagy modulation yields phase-dependent effects. During the acute ischemic phase, platelet autophagy is upregulated and contributes to microthrombosis, blood–brain barrier disruption, and inflammatory amplification [18,143,144]. Experimental inhibition of autophagic flux using chloroquine or hydroxychloroquine attenuates platelet aggregation and microparticle release under hypoxic conditions [145,146], suggesting that transient suppression of stress-induced autophagy may reduce thromboinflammatory injury during early ischemia.

In contrast, during reperfusion or chronic hypoxic adaptation, autophagy activation may support mitochondrial integrity and vascular repair [108,147]. Agents such as trehalose, Tat-Beclin-1 peptide, or rapamycin enhance autophagic flux and have been shown to reduce stroke susceptibility and preserve vascular function in experimental models [148]. However, excessive activation during reperfusion may exacerbate tissue injury, highlighting the importance of dosage and timing.

Collectively, these findings underscore the highly context-dependent nature of autophagy modulation in ischemic stroke. However, it is important to recognize that the majority of evidence derives from preclinical animal models or in vitro platelet systems. Direct clinical data specifically linking platelet autophagic flux to stroke severity, infarct progression, or therapeutic response remain limited. Moreover, most pharmacological modulators investigated in experimental stroke models exert systemic effects across multiple cell types, including neurons, endothelial cells, and immune cells, making it difficult to isolate platelet-specific contributions. Therefore, while autophagy-targeted strategies represent a compelling conceptual approach, their translation into clinically actionable platelet-directed therapies will require rigorous validation, precise temporal control, and careful assessment of bleeding risk and off-target consequences.

### 5.3. Potential for Combination Therapies

Integrating autophagy-targeting strategies with conventional antiplatelet therapies offers a promising avenue to enhance efficacy in thromboinflammatory conditions, particularly in patients with suboptimal responses to standard treatments. For example, clopidogrel resistance [149], a common issue in patients with diabetes, obesity, and chronic inflammation, may be addressed by co-administering autophagy modulators such as rapamycin or metformin. Metformin activates AMP-activated protein kinase (AMPK), stimulates autophagy, and improves mitochondrial function in platelets, thereby potentially restoring responsiveness to clopidogrel and reducing thrombotic risk.

Meanwhile, in the prevention of IS or MI, combining low-dose autophagy enhancers (e.g., spermidine or resveratrol) with aspirin may provide additive antithrombotic benefits without significantly increasing bleeding risk. However, careful dose optimization is required, as even low-dose aspirin carries a modest but non-negligible risk of hemorrhagic stroke. Enhancing autophagy too aggressively could exacerbate this risk, particularly in elderly or frail patients.

Moreover, dual-function compounds that modulate autophagy while exerting antioxidant or anti-inflammatory effects, such as polyphenols, may offer broader benefits in managing thromboinflammatory diseases. These agents not only improve metabolic profiles but also attenuate platelet activation, endothelial dysfunction, and vascular inflammation, making them attractive candidates for multimodal intervention in thromboinflammatory diseases [150].

Taken together, autophagy-based combination therapies represent a physiologically versatile and mechanistically diverse approach to platelet modulation (Table 1). When strategically paired with conventional agents, they may address resistance profiles, reduce oxidative stress, and lower thrombotic burden in a more tailored and patient-specific manner.

Although platelet autophagy represents an attractive intracellular therapeutic target, several important considerations temper its immediate translational promise. Unlike conventional antiplatelet agents that directly block surface receptors such as P2Y_12_ or integrin αIIbβ3, autophagy modulation operates upstream at the level of metabolic and stress response signaling [9]. While this intracellular positioning offers the theoretical advantage of restoring platelet homeostasis rather than bluntly suppressing activation, it also introduces complexity. Autophagy pathways are highly conserved and ubiquitously expressed across multiple cell types, raising concerns regarding systemic off-target effects [16]. Moreover, the dual and context-dependent role of autophagy implies that inappropriate timing or dosing could paradoxically increase thrombotic risk or exacerbate bleeding.

At present, most supporting evidence derives from genetic models or preclinical pharmacological studies, and clinical trials specifically designed to modulate platelet autophagy remain lacking. Therefore, while autophagy-targeted strategies may complement conventional antiplatelet therapies, particularly in metabolically driven or inflammation-associated thrombosis, their implementation will require precise patient stratification, validated biomarkers of platelet autophagic flux, and rigorous evaluation of safety in high-risk populations.

## 6. Challenges and Future Perspectives

**Figure 6:**
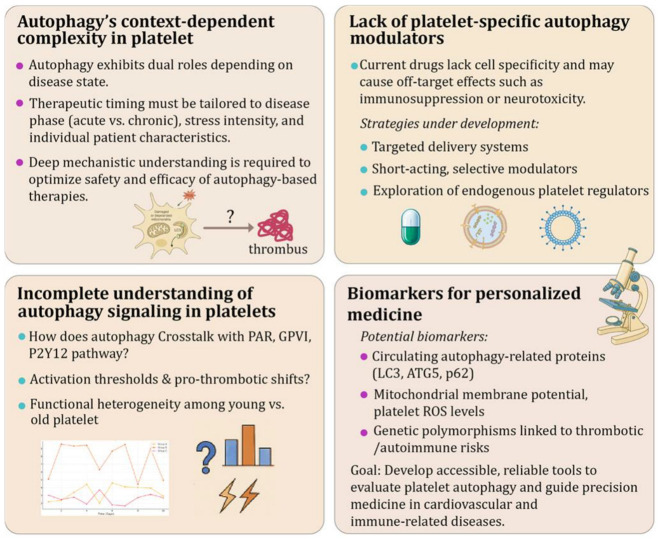
Challenges and Future Perspectives in Platelet Autophagy-Targeted Therapies. This figure summarizes the major barriers to translating platelet autophagy research into clinical applications. Key challenges include the dual and context-dependent roles of autophagy in platelet biology, the lack of platelet-specific autophagy modulators, limited mechanistic understanding of autophagy signaling pathways in platelets, and the absence of reliable biomarkers for clinical monitoring. Addressing these gaps will be essential for developing safe, targeted, and disease-tailored autophagy-based interventions in thromboinflammatory and autoimmune conditions.

Although autophagy is increasingly recognized as an important regulator of platelet function, turning this knowledge into effective therapies is still in its early stages. Key challenges remain, including the complexity of autophagy’s role in different disease settings, the lack of platelet-specific drugs, and limited tools to measure autophagy in clinical practice. To move forward, future research must focus on better understanding how autophagy behaves in various conditions, developing more precise treatment strategies, and identifying reliable biomarkers to guide therapy. The following sections highlight the main obstacles and opportunities in this emerging area (Figure 6).

### 6.1. Complexity and Context-Dependence of Autophagy in Platelets

A key challenge in developing autophagy-based therapies for platelet-related diseases is the context-dependent and dual nature of autophagy. Unlike conventional signaling pathways with largely unidirectional outcomes, autophagy in platelets exhibits dual effects, serving both protective and pathological functions depending on the physiological or disease context.

For example, enhancing autophagy may be beneficial in conditions marked by oxidative stress or metabolic dysfunction, where it helps clear damaged mitochondria and reduce reactive oxygen species. In contrast, in acute thrombotic events, autophagy may help sustain platelet activation and survival, potentially worsening thrombosis. In such scenarios, transient or context-specific inhibition of autophagy could be therapeutically advantageous. However, the precise thresholds at which autophagy shifts from protective to pro-thrombotic remain poorly defined.

To address this complexity, future therapeutic strategies must be precisely timed and tailored to the specific disease context. Key considerations include the severity and duration of platelet stress, the phase of disease progression (acute vs. chronic), and patient-specific factors such as age, comorbidities, and baseline autophagy capacity. Ultimately, a more detailed mechanistic understanding of how autophagy integrates into platelet signaling networks under distinct pathophysiological conditions will therefore be critical for optimizing both safety and efficacy of autophagy-directed interventions.

### 6.2. Lack of Platelet-Specific Autophagy Modulators

Most current autophagy modulators, such as rapamycin, metformin, and chloroquine, affect a broad range of cell types, including immune cells, endothelial cells, and neurons. This lack of specificity poses a significant challenge for therapeutic use, as systemic modulation of autophagy can result in off-target effects such as immunosuppression, impaired wound healing, or neurotoxicity. For example, although chloroquine can inhibit autophagy and attenuate platelet activation under hypoxic conditions, its widespread biological activity limits its feasibility as a platelet-directed therapeutic strategy.

To overcome these limitations, several strategies are being explored:

**Develop Platelet-Targeted Delivery Systems**: As autophagy-targeting therapies continue to evolve, emerging drug delivery platforms such as nanoparticles, platelet-specific liposomes, and ligand-conjugated systems offer promising opportunities for selective autophagy modulation [151]. These technologies could enable targeted delivery of autophagy modulators directly to activated platelets [152], thereby enhancing efficacy while minimizing systemic toxicity or off-target effects on vascular or immune cells. For example, platelet membrane-modified exosomes have been engineered to deliver therapeutic agents to atherosclerotic plaques, demonstrating enhanced specificity and therapeutic efficacy in preclinical studies [152,153]. However, the scalability, pharmacokinetics, and long-term safety of these delivery strategies remain to be fully established.

**Engineer Short-Acting, Selective Autophagy Modulators**: Designing agents with transient activity or restricted tissue distribution may help minimize unintended systemic effects. Advances in targeted drug activation strategies, including photoactivatable or ligand-directed systems, illustrate the conceptual feasibility of cell-specific autophagy regulation. For instance, in cancer research, PD-L1-targeting autophagy modulators have been developed for localized photoimmunotherapy, illustrating the feasibility of cell-specific autophagy regulation [154].

**Identify Endogenous Platelet-Specific Regulators**: Investigating intrinsic regulators of autophagy in platelets may offer novel therapeutic entry points. For example, emerging evidence linking sphingolipid metabolism to platelet autophagic regulation suggests a potential platelet-relevant axis for intervention [155]. Further mechanistic validation will be necessary to determine whether such pathways can be safely and selectively manipulated.

Overall, the development of platelet-specific autophagy modulators remains a critical prerequisite for clinical translation. Without improved targeting precision, systemic autophagy modulation may pose unacceptable safety risks in thromboinflammatory disease management.

### 6.3. Incomplete Understanding of Autophagy Signaling in Platelets

Despite recent progress, our understanding of the upstream regulators and downstream consequences of autophagy in platelets remains incomplete. Several key molecular questions need to be addressed to fully harness autophagy as a therapeutic target.

**Crosstalk with Platelet-Specific Signaling:** Platelets respond to various agonists, such as thrombin via Protease-activated receptors (PARs), collagen via GPVI (Glycoprotein VI), and adenosine diphosphate (ADP) via P2Y12, but how these classical signaling pathways intersect with autophagy remains unclear. For instance, does thrombin influence the activity of core autophagy regulators like ULK1 or Beclin1? Do second messengers such as calcium or DAG modulate autophagosome formation? Determining whether autophagy serves as a downstream effector or upstream modulator in these pathways is critical for understanding its regulatory role.

**Thresholds and Triggers of Autophagy Activation**: The specific cellular cues that trigger autophagy in platelets, such as energy depletion, mitochondrial dysfunction, or oxidative stress, have not been well defined. Moreover, the thresholds at which autophagy shifts from a protective role to a pro-thrombotic driver remain unknown. Defining these context-specific transitions is essential for designing appropriately timed therapeutic interventions.

**Autophagic Heterogeneity Across Platelet Subpopulations**: Platelets are a heterogeneous population, with young (reticulated) and aged platelets differing in function and metabolic activity. Whether these subtypes exhibit distinct autophagic profiles, such as differences in LC3-II expression, mitophagy capacity, or lysosomal function, has not been systematically studied. Such heterogeneity may explain inter-individual differences in thrombotic risk and drug responsiveness.

Addressing these mechanistic uncertainties will require advanced experimental strategies, including platelet-specific CRISPR/Cas9 models, real-time assessment of autophagic flux, and integrated single-cell and bulk multi-omics approaches.

Beyond therapeutic modulation, platelet autophagy may also have implications for platelet storage and transfusion practice. During storage, platelets undergo progressive metabolic and mitochondrial alterations, collectively termed the “storage lesion”, characterized by oxidative stress, bioenergetic decline, and functional impairment [27,156]. Emerging observations suggest that dysregulated autophagic flux may contribute to these storage-associated changes [27], although systematic evaluation of LC3-II/LC3-I dynamics and flux markers in stored or post-transfusion platelets remains limited. Understanding how autophagic integrity influences post-transfusion platelet survival and functionality may offer opportunities to optimize storage conditions and improve clinical outcomes.

Collectively, resolving these mechanistic and translational uncertainties is essential for translating platelet autophagy biology into precise, mechanism-informed interventions for thrombotic, inflammatory, and immune-related disorders.

### 6.4. Biomarkers for Personalized Medicine

To translate autophagy-modulating therapies into clinical practice, there is a pressing need for reliable biomarkers that can guide personalized treatment decisions. As platelet autophagy exhibits context-dependent effects, identifying patients who are most likely to benefit from either enhancing or suppressing autophagy is critical.

**Circulating Autophagy-Related Markers**: Proteins such as LC3, ATG5, and p62 may serve as indirect plasma markers of autophagic activity. Shifts in their levels have been linked to changes in autophagic flux [157]; however, their specificity for platelet-derived autophagy remains to be validated in clinical settings.

**Mitochondrial and ROS Markers in Platelets**: Given autophagy’s role in maintaining mitochondrial integrity, assessing mitochondrial membrane potential, ROS levels, and related metabolic indicators in circulating platelets may provide useful functional readouts of autophagy status and individual thrombotic risk.

**Genetic and Multi-Omic Signatures**: Genetic polymorphisms in autophagy-related genes (e.g., *ATG16L1*, *IRGM*) have been associated with thrombotic and autoimmune diseases [158,159]. Integrating such variants with inflammatory and metabolic markers could enhance risk prediction and patient stratification, supporting more personalized treatment approaches.

Despite these promising directions, standardized and platelet-specific biomarkers for assessing autophagy in clinical samples are still lacking. Future efforts must focus on developing accessible tools that can link autophagy status to clinical outcomes, thereby enabling precision-guided use of autophagy-targeted therapies in cardiovascular and immune-mediated disorders.

## 7. Conclusions

Autophagy, once considered irrelevant in anucleate platelets, has emerged as a vital regulatory mechanism of platelet function, bridging cellular homeostasis and pro-thrombotic activity. The presence of a complete and functional autophagic machinery in platelets not only supports their metabolic integrity and survival but also modulates their activation, aggregation, and response to pathophysiological stress. These insights redefine our understanding of platelet biology and open new avenues for therapeutic intervention.

In thrombosis, autophagy plays a context-dependent dual role, supporting platelet function under physiological conditions while restraining excessive activation during metabolic, oxidative, or inflammatory stress. In disease states such as ischemic stroke, cardiovascular disorders, and autoimmune conditions, dysregulated platelet autophagy contributes to disease progression by altering platelet reactivity, survival, and interactions with the immune and vascular systems.

Therapeutically, targeting autophagy offers a promising intracellular strategy to modulate platelet function beyond traditional surface receptor antagonists. Both autophagy inducers and inhibitors have demonstrated potential benefits, though their effects are highly dependent on disease context, timing, and individual variability. The clinical translation of this approach remains limited by the lack of platelet-specific modulators, validated biomarkers, and tools for dynamic monitoring of autophagic flux. However, progress in targeted drug delivery, real-time imaging, and systems biology is gradually overcoming these hurdles. By bridging basic mechanistic insights with disease relevance, this review highlights autophagy as a novel and versatile regulatory axis in platelet biology. Autophagy-targeted strategies may ultimately complement or refine current antiplatelet therapies, offering a more precise and adaptable approach to managing thrombotic and immune-mediated platelet disorders.

## Figures and Tables

**Table 1:** Autophagy-Based Therapeutic Strategies for Platelet Dysfunction Across Disease Contexts.

Disease Context	Specific Disease/Condition	Autophagy Status in Platelets	Therapeutic Agent	Mechanism of Action	Therapeutic Outcomes
Metabolic Syndrome					
	Type 2 Diabetes/CAD	Impaired autophagy, reduced mitophagy, increased ROS	Metformin	AMPK activation, enhances mitophagy, reduces ROS	Improves platelet function, lowers cardiovascular risk
			Spermidine	SIRT1/AMPK mTOR axis modulation, restores autophagy	Reduces oxidative damage, improves vascular repair
			Resveratrol	AKT/mTOR modulation, antioxidant and anti-aggregatory	Restores autophagy, inhibits platelet aggregation
Autoimmune Disorders					
	Immune Thrombocytopenia (ITP)	Suppressed autophagy, increased platelet apoptosis	Rapamycin/ABO	mTOR inhibition, restores LC3-II, protects mitochondria	Increases platelet survival, effective in refractory ITP
	Systemic Lupus Erythematosus (SLE)	Elevated autophagic activity	Hydroxychloroquine (HCQ)	Lysosomal inhibition, TLR suppression, immune modulation	Reduces platelet activation and inflammation in SLE
	Antiphospholipid Syndrome (APS)	Elevated autophagic activity	Hydroxychloroquine (HCQ)	Lysosomal inhibition, TLR suppression, immune modulation	Decreases thrombotic risk in APS via immune modulation
Acute Ischemic Stroke/Hypoxic Conditions					
	Acute phase of ischemia or hypoxia	Upregulated autophagy during the acute phase	Chloroquine/HCQs	Inhibits autophagic flux, reduces microparticle release	Prevents thrombus propagation and BBB damage in early stroke
	Chronically hypoxic condition	Upregulated autophagy during reperfusion	Trehalose/ Tat-Beclin 1 peptide	Enhances autophagic flux, improves mitochondrial quality	Enhances mitochondrial integrity, reduces stroke susceptibility
	Chronically hypoxic condition	Upregulated autophagy during reperfusion	Rapamycin	Activates autophagy, preserves vascular function	Limits reperfusion injury, supports BBB and neuronal recovery

## Data Availability

No new data were created or analyzed in this study. Data sharing is not applicable to this article.

## Abbreviations

The following abbreviations are used in this manuscript:

AMPK	AMP-activated protein kinase
APS	Antiphospholipid syndrome
ATP	Adenosine triphosphate
BBB	Blood–brain barrier
CVD	Cardiovascular disease(s)
HCQ	Hydroxychloroquine
IS	Ischemic stroke
ITP	Immune thrombocytopenia
LC3	Microtubule-associated protein 1 light chain 3
MI	Myocardial infarction
mTOR	Mechanistic target of rapamycin
PI3K	Phosphoinositide 3-kinase
ROS	Reactive oxygen species
SLE	Systemic lupus erythematosus
